# A Treatise on the Diseases of the Skin

**Published:** 1834-10-01

**Authors:** 


### A Treatise on the Diseases of the Skin. By *P. Rayer*, D.M.P. Translated from the French, by *W. B. Dickinson*, Octavo, pp. 398. London, 1833.

The nosology of cutaneous affections has been one of the most puzzling problems in medical science; and perhaps there is no set of diseases, of which the classification is so far from being determinate and satisfactory, even at the present day. It would not be necessary to allude to the thousand and one errors which were partly caused, and partly increased, by the adoption of a vague and most unscientific nomenclature, were it not that our Continental brethren have almost uniformly, until the appearance of Rayer's admirable work, retained some of its most annoying absurdities; witness the manifold and incongruous meanings of those words, "teigne" and "dartre," not only in the writings of Alibert, but also in those of most of the best French authors. We are told that it "was common to designate under the former name all chronic inflammations of the hairy scalp, and, under the latter, all chronic inflammations, and some acute diseases, of the face, the trunk, and the limbs." Not to speak of the absurdity of giving different names to the same disease, according to the part of the body where it appears (as well might a botanist make different species of a plant from its being found on walls, or in meadows, or in woods), let us glance at the subdivisions of the generic "dartre;" Alibert enumerates seven species, viz. the crustaceous, erythemoid, furfuraceous, phlyctenoid, pustular, corroding, and the squamous—such a confused hotch-potch of contrary things! By far the most rational attempt at the classification of skin diseases, previous to the publication of Willan's immortal work (which we are ready to confess was indebted, in some respects, to its predecessor), was Plenck's "Doctrina de Morbis Cutaneis," printed in 1796: he formed 14 classes—maculæ, pustulæ, vesiculæ, bullæ, papulæ, crustæ, squamæ, callositates, excrescentiæ, ulcera, vulnera, insecta cutanea, morbi unguium, morbi capillorum. Willan very properly struck out several of these divisions, and reduced them to eight; and so satisfied has the profession been with the superiority of the arrangement proposed by him, that, with but a few exceptions, it has been universally adopted, and with great propriety; for the main errors which it is possible to charge against it, seem almost inseparable from the nature of the subject; many of the diseases being of so Proteiform a character, that they defy any simple and exclusive arrangement in a nosological catalogue. The correctness of this remark seems amply warranted, by the very results of the attempts made by Rayer and others, to amend what was deemed faulty or defective in Willan's system. We agree with them in some of their objections to particular parts of it; purpura hæmorrhagica ought not, perhaps, to be associated with measles among the exanthemata—pemphigus and pompholix need not have been separated—scabies or psora is oftener a papular or a vesicular, than a pustular disease—acne and sycosis belong much rather to the class of pustules than of tubercles; and there is no notice taken of the morbid changes of the hair, nails, &c. All this is true, and we admit that some modifications (especially the retrenchment of many of the species) may, with advantage, be introduced into Willan's arrangement; but it still is, and in our opinion will long continue to be, the guide of medical men, in investigating the diseases of which it treats. Plumbe and Rayer, who have commented upon its imperfections, have each proposed a classification of their own: with that of the former, founded partly upon anatomical, partly upon physiological, and partly, also, upon pathological speculations, we mean not to deal; the profession, while approving of the "Practical Treatise on the Diseases of the Skin," has not, and we think justly, adopted the arrangement which the author has employed; we shall, therefore, proceed to lay before our readers that on which the work now under review is founded. The following synoptical table presents us with a view of the—

### CLASSIFICATION.

### SECTION I.

DISEASES OF THE SKIN.

CHAPTER I. *Inflammations of the Skin.*

1°. *Exanthematous:* Rubeola, roseola, scarlatina, urticaria, erythema, erysipelas.

2°. *Bullous:* Vesication, ampullæ, pemphigus, rupia, zona.

3°. *Vesiculous:* Herpes, psora, eczema, miliaria.

4°. *Pustulous:* Varicella, variola, vaccina, vaccinella, ecthyma, cuperosa, mentagra, impetigo, tinea, artificial pustules.

5°. *Furunculous:* Hordeolum, furuncle, anthrax.

6°. *Papulous:* Strophulus, lichen, prurigo.

7°. *Tuberculous:* Lupus, cancer, elephantiasis of the Greeks.

8°. *Squamous:* Lepra, psoriasis, pityriasis.
9°. *Linear:* Fissures.
10°. *Gangrenous:* Malign pustule, carbuncle of the plague.
11°. *Multiform:* Burns, frost-bite, syphilitic eruptions.

| | |
|---|---|
| CHAPTER II. *Cutaneous and Subcutaneous Congestions and Hæmorrhages.* | Cyanosis, vibices, petechiæ, purpura hemorrhagica, ecchymosis, dermatorrhagia. |
| CHAPTER III. *Neuroses of the Skin.* | Exaltation, diminution, abolition of the sensibility of the skin, without appreciable alteration in the texture of this membrane. |
| CHAPTER IV. *Alterations in the Colour of the Skin.* | *Decoloration*: Leucopathia (Partial, General); Chlorosis. *Accidental Colorations*: Ephelis, lentigo, chloasma, meladermis, icterus, *nævus maculosus,* bronze tint produced by the internal use of lunar caustic. |
| CHAPTER V. *Morbid Secretions.* | Ephidrosis, acne,* folliculous tumors. |
| CHAPTER VI. *Defects of Conformation and Texture; Hypertrophies, & Accidental Productions.* | Distention of the skin; cicatrices, vegetations, nævus hematodes, subcutaneous vascular tumors; warts, pearly granulations; corns, icthyosis, horny appendages. |

### SECTION II.

### ALTERATIONS OF THE APPENDAGES OF THE SKIN.

| | |
|---|---|
| CHAPTER I. *Alterations of the Nails, and of the Skin which produces them.* | Onyxis; increased growth of the nails; spots, change of colour, fall, desquamation, reproduction, &c. of the nails. |
| CHAPTER II. *Alterations of the Hair, and of the Follicles which produce it.* | Inflammation of the bulbs of the hair; accidental coloration, canities; alopecia; matting of the hair; plica; accidental pilous tissue. |

### SECTION III.

### FOREIGN BODIES OBSERVED ON THE SURFACE, OR IN THE SUBSTANCE OF THE SKIN.

| | |
|---|---|
| *Inanimate.* | Dirt, dirt of the scalp of new-born children; inorganic matters, artificial colorations. |
| *Animate.* | *Pediculus, humani corporis; P. capitis; P. pubis: pulex irritans, P. penétrans; acarus scabiei; œstrus; gordius.* |

---

* "This is the acne *punctata* of Bateman; the French term, *tannes,* is hardly to be rendered into English; *grub,* I believe, is the vulgar term for this affection.—T."

### SECTION IV.

### DISEASES PRIMARILY FOREIGN TO THE SKIN, BUT WHICH SOMETIMES PRODUCE PECULIAR ALTERATIONS IN THIS MEMBRANE.

Elephantiasis of the Arabs."—*Introduction, p.* xxvii.

Our remarks must necessarily be limited to the first and fourth sections—the second and third, it will be observed, appertain to the appendages of the skin, and may, therefore, at least for our present consideration, be excluded, especially as the title-page of the work professes to treat only of the "Diseases of the Skin."

The first impression left on the mind, from an examination of this classification, is the analogy which exists between its principles and those of the nosology of Cullen; we have the inflammations, or phlegmasiæ—the hæmorrhages—the neuroses, or nervous diseases—and the other three chapters might, with some propriety, be classed under the general term of cachexiæ. We may, therefore, presume that several, at least, of the objections which have been urged against the one, are not inapplicable to the other. We do not, it must be obvious, mean to say that the orders of Rayer are exactly parallel to, or correspond with, those of Cullen—the first glance must satisfy us that this is not the case; the lepra of the one is a phlegmasia, and a cachectic impetigo of the other; the psora of Cullen is most preposterously arranged as a local dialysis, or, in other words, a broken surface, whereas it is a vesicular inflammation of Rayer; but the principle which has led the latter to refer so many and so different diseases to his first chapter, is essentially the same which prompted the former to call diarrhœa and diabetes nervous diseases, and to attribute all the various forms of dropsy to a cachectic state of the constitution; in short, theory is the basis of a large portion of both systems, and is the pervading error in both. There is no difficulty in adducing a score of proofs, to shew how largely imbued with theory the system of Rayer is—take, for example, the eighth order, the squamous inflammations of the skin: it comprises three diseases, lepra, psoriasis, and pityriasis; now, not to allude to some of the varieties of the two former diseases, it is surely giving an almost illimitable extension to the word inflammation, to apply it to the majority of cases of pityriasis. Are they attended with pain, heat, redness and swelling? Assuredly not; and although the itching be sometimes troublesome, yet we cannot bring our minds to the conclusion, that therefore there must be some degree of inflammatory action present. Then, again, that singular disease, icthyosis, is banished from this order, because we are told "that it is almost always congenital, or developed in the first few months after birth, and is not attended either by sanguineous injection of the skin, morbid heat, itching, or by any other sign of inflammation;" the same may be said, however, of many old cases of lepra itself, and the circumstance of nearly the same treatment being found serviceable in both diseases adds not a little probability, that their proximate or essential causes are somewhat allied.

That most distressing malady, "prurigo," may also be adduced, as another instance of the extreme latitude which it is necessary to give to our idea of inflammation, if we are to assent entirely to Rayer's classification. The skin is often, nay generally, as pale as in health; there is frequently little or no elevation of the surface; there is certainly no pain, although there may be an intolerable itching: the disease, indeed, appears to be one which affects the cutaneous nerves, much rather than the cutaneous blood-vessels, and it may be, therefore, referred, with quite as much propriety, to the order neuroses as to that of phlegmasiæ. Here, too, the appropriate treatment, at least in a vast number of cases, is the very reverse of the antiphlogistic, unless, indeed, we are to reckon stimulating lotions, bark and other tonics, opium, and so forth, as such.

If the preceding instances shew that not a little compulsion, nay, absolute violence, must be used, to force some of the members of Rayer's inflammatory diseases into his first section, what shall our readers say when they turn their eyes to the ninth order, which has received the classic name of "linear inflammations," vulgarly denominated "chaps," or cracks of the skin—and when they find that chapped feet, ulcerated nipples, the fissures in the skin, so common in dropsy and during pregnancy, have all, more or less, the features of inflammatory action. Then, again, is it right that the Proteiform class of eruptions, which succeed to primary syphilis, are classed among inflammatory diseases? Certainly not—if we are to attach the ordinary meaning to the word inflammation; for they neither have, at least in a multitude of cases, the outward and visible signs of increased action, nor are they attended with the constitutional symptoms of active pyrexia, and lastly the successful mode of treatment consists rather in strengthening the powers of the system than in lowering them. These arguments cannot, in our judgement, be gainsayed, or answered, and afford sufficient grounds for withholding our entire assent to the classification of Rayer, in all its details. If there was need of any more, we might allude to the exclusion of the various forms of acne, and of follicular tumors from the first section, and their insertion in the fifth. True it may be, that in these diseases the secretions of the skin may be morbid; we do not deny it; but the simple question which we propose is, whether there is no attendant inflammation at the same time; and if there is, how comes it that they are not classed with the other inflammations. One other remark upon the arrangement and we have done.

It may be fairly doubted, whether the elephantiasis of the Greeks, the elephantiasis of the Arabians, and the production of what have been called "mamillated excrescences" on the skin, ought to be viewed as diseases so widely different from each other as to require that all three be put into different sections of the code. Our knowledge indeed of the morbid changes connected with the diseases now mentioned, is, it must be confessed, far too imperfect to warrant us in giving a decided opinion either way; but we think that it would have been wiser to have brought them nearer together, with the view, had there been no other reason, to compare and contrast them together.

We had almost forgot to allude to the capricious arrangement of the orders in the first section; ought not the papulous diseases to precede the bullous, vesiculous, pustulous and tuberculous, for the very good reasoning, that these four last forms of cutaneous disease very generally exhibit a papulous appearance at first? and again, two of the members of the tuberculous order seem very much akin to those of the ninth or gangrenous.

Such are some of the objections which may be urged against our author's method of classifying the diseases of the skin; and we are the more anxious to direct the attention of our readers to the consideration of what has been stated above, as the prevailing doctrine of the whole work, not only in regard to the nosology proposed, but equally so, to the treatment recommended, is, that inflammation in some form, part, or organ, is an almost invariable and necessary accompaniment of cutaneous eruption. Every page of the work before us bears testimony that Rayer is a most fond admirer of the Broussaist creed; we think, extravagantly so; because, while we admit that the "Physiological Physician" of the Val de Grace, deserves well of the profession for having introduced a more rigorous and comprehensive method of examining all the symptoms of a disease, and for having disabused our minds of many of the phantom dangers of debility, he seems to us to have overstepped the bounds of legitimate deduction, and to have inferred the existence of his "favourite disease," although neither indicated by symptoms during life, nor verified by necroscopic inquiry. The mischievous effects of ultra-Broussaism may not indeed be so great as those of an opposite system; but both are bad; and bad, because they are exclusive. The practice of a physician attaching himself to one particular doctrine, which is made applicable to all diseases, and to all circumstances of disease, arises, we fear, too often from mere selfish and mercenary motives; the love of notoriety, and the ambition of success are dangerous enemies to quiet thought and cautious reasoning; and when to these impulses is added the obstinacy of wounded pride, or of audacious conceit, or of arrogant ignorance, we need not wonder that strict veracity is sometimes sacrificed to pompous display. Now, we do not mean to impeach the good faith of M. Broussais and of his disciples; but we must certainly question their candour or clear-sightedness. They cannot possibly have failed to observe, and if so, they ought to acknowledge, that patients labouring under their supposed gastro-enteritis have in a thousand instances speedily recovered under the use of occasional aperients and of a light bitter tonic, after having been subjected to leeching, and leeching, and leeching, and to all the ills of a most rigid "diete methodique." We again confess that benefit, decided benefit, has been derived from the more scrutinizing method of examining disease introduced of late years by the physiological physicians of Paris; for the grand aim and object of a skilful practitioner will ever be to arrive at a correct diagnosis of his patient's case; and the knowledge of this cannot be acquired except by a diligent and most inquisitive examination of all the leading functions of the body; of sensation, of motion, of digestion and assimilation, of respiration and so forth; and if this be uniformly done, with a mind unfettered by the opinions of others, and unprepossessed with any favourite doctrines, we venture most unequivocally to assert, that no exclusive system of therapeutics will be received as all-curing or catholic.

The "purgez a droit" and "saignez a gauche" practice, must therefore be deprecated, at least to the extent to which it has been carried within the last five and twenty years. The weapons of ridicule and satire are often more effectual in conquering prejudice and error, than those of grave reasoning, and we doubt not that the wit of a Le Sage or of a Moliere could, within the compass of a single page, most triumphantly annihilate the many goodly volumes which have so long been received as gospel truth by a wondering profession. We have been led to make these few remarks by the perusal of the work before us; we think that it is too largely imbued with one doctrine throughout; a doctrine, which however useful in many of its precepts, is apt to be carried to an extravagant extent, and to lead to a practice often most unsatisfactory, and sometimes decidedly pernicious; for example, we are told that a stye on the eyelid is very generally indicative of internal inflammation; "Hordeolum almost always coincides with gastro-enteritis." Surely this is what Sterne would have called "rather too hobby-horsical." Indeed the author himself seems on one occasion to be aware of this; for at page xvi. of the introduction (which by the bye is a most admirable essay, and in itself would render the present work well worthy of study) we find it stated—

"Up to the present time but a very small number of anatomical researches have been made on diseases of the skin. Among the authors who have seen and pointed out the importance of them, there are some who have given them a false direction, in pointing their attention principally to internal lesions of the lungs, digestive organs, and uterus, which have taken place accidentally in individuals attacked by chronic diseases of the teguments; but that these observations have been of some utility, is shown by their having proved, by numerous examinations of the body, that these internal lesions more frequently coincide with inflammations of the skin, than several other affections which may be equally complicated with them. New anatomical researches on the structure of the alterations of the teguments have furnished me with valuable characters for the distinction of various forms of phlegmasiæ; and I have given, in the history of rubeola, erysipelas, variola, tinea favosa, purpura hemorrhagica, onyxis, icthyosis, &c. some anatomical details which have not perhaps been hitherto published with equal accuracy."—*Introd.* xvii.

We quite coincide in the correctness of these opinions. Before proceeding to the examination (necessarily a short one) of the contents of this volume, the following remarks on the delineation of skin diseases are so just, that we cannot do better than extract them.

"Some authors have endeavoured to make their descriptions of these diseases more striking by the aid of coloured figures: the two most beautiful collections of this sort are, without exception, those of Willan and Bateman,* and of Alibert.† Most of these plates exhibit, however, only one of the stages of the inflammation which they are intended to represent. Thus it happens, that some diseases described as *pustulous* have been exhibited in the *squamous* state; that some others also, placed among the *pustulæ*, have been represented in the *tuberculous* state; that others, lastly, have been only drawn in the state of *crusts*, a secondary character, and common to several very different forms of *phlegmasiæ*. These faults I have endeavoured to avoid in arranging the plates of this work.‡ The primary forms of the inflammations of the skin, and the alterations which succeed them, have been represented carefully after nature, or the best engravings that have been published.

Figures of the natural size, in which the head, trunk, limbs, or whole body, are represented, would be out of place in an elementary work; while, on the

---

* Bateman (Th.) *Delineations of Cutaneous Diseases*, 4to. London, 1817.

† Alibert. *Op. cit.*

‡ Mr. Dickenson has committed the egregious blunder of frequently referring to particular plates, attached to Rayer's work; and yet we find that he has not published any of them.

other hand, they would have only the trifling advantage of exhibiting the same alterations in a greater number of points: a hundred pustules, or twenty tubercles of cuperosa, for example, scattered over the face of a woman, with a design of the head and bust given, and additions more less elegant, do not yield a juster idea of the pustulous form of this affection, and of the tubercles by which it sometimes terminates, than does a piece of skin on which these alterations are shewn. Lastly, some papulæ of prurigo or lichen, a few squamous plates of lepra or psoriasis, &c. suffice to make known the character of these diseases, without its being necessary to represent all the regions of the body upon which they have been observed."—*Introd.* xviii.

The first order, the exanthematous, comprises six genera, five of which belong to Willan's exanthematous order, viz. rubeola, roseola, scarlatina, urticaria, erythema, and erysipelas. Rayer is decidedly right in excluding purpura from this order, and in admitting erysipelas, which Willan strangely enough separated from erythema—only a milder form of itself.

"The red tint produced by the effusion of blood into the subcutaneous cellular tissue or skin, differs so much from that of the exanthemata, that we are at a loss to conceive how two such observant men as Willan and Bateman could see any resemblance between petechiæ, purpura hæmorrhagica, and the exanthemata. In the latter the red tint is obscure, and the diagnosis more difficult in negroes than in whites.

Exanthemata are sometimes complicated with other, and particularly with papulous, vesiculous, and bullous inflammations of the skin. Thus, intense erysipelas, abandoned to itself, is frequently surmounted by bullæ similar to those of pemphigus. In this point of view, it seems to form an intermediate link between exanthematous and bullous inflammation." 9.

The general scope of treatment, in the exanthemata, is well and clearly stated in the following extract.

"They require the antiphlogistic treatment and diet. When caused by miasmatic poisoning, this treatment must be followed with more reserve. We must not expect to cut them short by repeated bleeding, as has been advised by some pathologists. It is important also to keep in mind, that inflammations of the gastro-pulmonary mucous membrane present a prominent feature in exanthematous diseases of the skin, and that it is to this, above all other complications, that the attention of the practitioner should be directed. The moment symptoms of these internal phlogoses are observed, they should be narrowly watched, and, if intense, subdued; treating them nearly as if the cutaneous affection was not present. These gastro-pulmonary inflammations sometimes survive that of the skin. They require more cautious treatment during convalescence, as a careless regimen or injudicious medicine may aggravate them, and so become the cause of a more or less grave relapse." 9.

In the description of rubeola, we are told that this disease "differs from roseola, not only in the form of the exanthema, but also by the gastro-intestinal inflammation which accompanies it;" and yet, a few pages further on, it is stated that "this fugacious inflammation (roseola) is often connected with gastro-enteritis, which may either precede, accompany, or continue after it has disappeared;" and, to add to this confusion, in the very same page from which this latter extract is taken, we are informed how to distinguish the two diseases, thus:

"In rubeola, the eruption, usually preceded and accompanied by a gastro-bronchitis, takes place regularly the fourth day of a febrile affection; it is prolonged till the seventh or eighth, and during convalescence gastro-pulmonary inflammations, more or less severe, frequently supervene. In roseola the eruption appears without any obvious cause, or it may seem to depend on the existence of some other cutaneous inflammation, or on phlogosis of the gastro-pulmonary mucous membrane:" 19.

An apt illustration, among many others, of the indiscreet adoption, by our author, of the Broussaian doctrines. It has been a question among medical men, whether Willan is right in admitting, as distinct species of the genera rubeola and scarlatina, those forms of the diseases, in which either the exanthema is absent, while the other symptoms, such as the catarrh in the one case, and the affection of the throat in the other, are present; or in which the exanthema exists, unattended with these symptoms now mentioned—thus, authors mention a "rubeola sine catarrho," and "a scarlet fever without eruption." Rayer says—

"Most of those cases entitled *rubeola without catarrh,* and mentioned in some treatises, are really nothing more than cases of roseola or erythema. The presence of the exanthema prevents the disease from being confounded with pulmonary catarrh. Those pretended cases of *measles without eruption* would more properly be entitled to the name of *catarrh without measles:* for, even supposing that the gastro-pulmonary inflammations that are observed during epidemics of measles, to be produced by the specific cause of this latter, still they present no peculiar characters distinguishing them from ordinary phlegmasiæ of the air and digestive passages, or anything to authorize us to consider them as varieties of rubeola." 13.

These sentiments we consider to be too absolute; for there can be no doubt that, during the prevalence of an epidemic, either of measles or of scarlatina, it is by no means unfrequent to observe among the members of the same family, living together, and exposed to the same influences, some affected with the genuine disease, we mean that form in which both the internal and external symptoms are present, while others exhibit only one set of them. Now, under these circumstances, we consider it to be rather a stretch of incredulity, to deny the identity of the two affections; still it is possible that they are indeed different; and, perhaps, the only satisfactory method of deciding the question is, to ascertain whether those patients in whom only one set of the symptoms shew themselves, be as effectually secured against a return of the disease as the others. The question is not easily solved; but we can see no other way of doing so, but that we have now mentioned.

In the account given of the diagnosis between the exanthema of scarlatina, and that of roseola, it is laid down that—

"The tint of scarlatina is much brighter, and more permanent and uniform than that of roseola. In scarlatina the redness disappears on pressure, and takes place again from the circumference towards the centre of the finger-print, when the pressure is removed; while in roseola, the morbid coloration collects indiscriminately upon all the points which had been subjected to pressure." 20.

Is this distinctive character uniformly correct? Our own experience does not warrant us to decide. A much more important topic, appertaining to scarlatina, arrests our attention. So essentially inflammatory is this disease, in our author's opinion, that he hesitates not to affirm that, just in proportion to its severity and danger, so is the amount of inflammation present, and so, therefore, ought to be the activity of the antiphlogistic treatment. The narrative of the worst species, the scarlatina maligna, is thus very faithfully given.

"It comes on like scarlatina anginosa, and in the course of two or three days is characterised by symptoms of extreme gravity. The appearance of the exanthema is tardy, its colour feeble and livid, mixed with petechiæ, and its duration is uncertain. It may disappear and reappear several times. The pulse is small and irregular, the teeth and tongue are covered with brown or black crusts, the eyes are greatly injected and sight confused, the cheeks are of a deep-red colour; there exists also deafness and delirium in adults; coma and agitation in children; fetid breath, difficult laborious respiration, increased by the thick viscid mucosities deposited in the pharynx; deglutition is difficult or impossible, with constriction of the jaws, and a blackish exudation on the surface of the amygdalæ and neighbouring parts. A continual coma, difficulty of respiration, abundant diarrhœa, and formation of numerous petechiæ, announce approaching death. The few patients who survive these sufferings are then attacked by inflammations of the air-passages and digestive organs, which remain after the cure of the exanthema. Gangrenous eschars are often found over the trochanters and sacrum; they are followed by extensive ulcerations, the difficult cure of which lengthens convalescence. When associated with chronic cæco-colitis, these ulcerations are always dangerous, and sometimes fatal." 24.

This frightful form of the disease is occasioned, we are told, "by the simultaneous or successive inflammation of the pharynx, skin, stomach, intestines, larynx, and brain" (by the way, a strange mode of arrangement); and in the description of the treatment it is stated, that—

"In scarlatina *anginosa*, bleeding from the arm or foot, the application of leeches to the neck or epigastrium, mild sinapisms to the feet, emollient cataplasms to the throat, mucilaginous drinks, &c. are usually indicated. In scarlatina *maligna*, or, to use a more accurate expression, scarlatina complicated with intense inflammation of the pharynx, stomach, intestines, and bronchia, cerebral congestion, or arachnitis, &c. the antiphlogistic measures should be more active, according to the gravity of these different affections." 27.

Well may the author add, "we must not carry the bleedings so far as in themselves to become dangerous; and judgment is requisite here as well as in the treatment of other inflammations."

The above practice, in the extent recommended, we hesitate not to denounce as most murderous: it is a gross and fatal error to believe that the malignancy of this, and of other putrid diseases, is attributable solely to the extent or severity of the existing inflammation; and the consequences of its adoption have been quite as fatal as those ever caused by the humoral pathology. The only rational, and certainly the only safe doctrine upon which we can proceed in the treatment of such diseases, is one compounded partly of the Broussaian and partly of the humoral principles;—that the condition of the blood and of the secretions is materially altered (we speak neither of the nature nor of the cause of the alteration) even from the first, no one, who uses his eyes and will speak the truth, can for a moment deny; and equally true it is, that an inflammation, or congestion, or something akin to these morbid states, does exist at the same time in some parts or organs of the body. We challenge the most enthusiastic of either sect to gainsay either of these propositions; and yet, strange to tell, in spite of their reason, they almost wilfully shut their eyes to the truth, and make their practice belie their convictions. We again repeat, that the only rational view of the subject is to study all the symptoms, and to exclude none; to examine the blood, to examine the secretions, the state of every organ as far as we can, to discover, if possible, whether any be more oppressed or affected than another; to watch the effects of the remedies employed, and all the time to keep our minds from being swayed by any preconceived or pre-adopted theories. What may be termed the "eclectic" mode of treatment is therefore decidedly the best; viz. to apply leeches where congestion or inflammation is ascertained to exist, to exhibit salines or vegetable acids, to sponge the surface repeatedly with antiseptic lotions, such as the solutions of the chlorides, to regulate the secretions, and, if necessary, not to withhold stimulants, of which by far the best is ammonia—it is much preferable to any spirituous and vinous preparations. Should these last be necessary, it will be proper to exhibit them in effervescing draughts; and we avail ourselves of the present opportunity of strongly recommending a modification of these draughts, which we have been long in the habit of using with most satisfactory success in many malignant or putrid diseases. The addition which we make is 15 grains or a scruple of common salt, (muriate of soda,) and a small tea-spoonful of the spiritus ætheris nitrici. The draught should be repeated every two or three hours.

The chapter on erythema and erysipelas is exceedingly good, and merits commendation throughout, with the exception of the following sentence.

"Since Dessault, most French pathologists recommend the administration of an emetic at the outset of erysipelas. It produces a salutary effect when this affection has followed wounds of the head, but is dangerous when gastro-enteritis exists, the morbid phenomena of which have been designated collectively under the names of *bilious plethora, intestinal* or *gastric irritation, bilious fever,* &c., though in these cases it has been more especially recommended." 48.

*Bullous Inflammations.* On referring to the synoptical table at the commencement of this article, it will be observed that five diseases belong to this order. Willan has only three, viz. erysipelas, pemphigus, and pompholyx;—this is one of the most faulty parts of his system. Erysipelas, as we have already stated, belongs rather to the exanthemata; the bullæ being quite accidental, or, at least, by no means essential to the disease.—then again, pemphigus and pompholyx are assuredly not distinct diseases; the mere presence or absence of febrile symptoms is not sufficient to warrant Dr. Bateman in saying that they "differ most materially" from each other.

The transference of rupia from the order "vesiculæ" to that of the "bullæ" is of questionable propriety; and the very description which Rayer has given, applies much better to a vesicular than to a bullar disease. He says, "The bullæ are small, distinct, and flat, with inflamed bases, containing a transparent humour, which gradually becomes turbid, puriform, and the bullæ are transformed into thick, chocolate-coloured crusts, from the desiccation of the serous sanguinolent fluid." Sometimes indeed these crusts become large and very prominent, so as to resemble limpet-shells; but they are very different from the dried bullæ or bladders, as seen in pemphigus. Rupia appears to be a disease intermediate between the vesicular and the pustular orders; it has, moreover, many points of affinity to ecthyma; they both occur under similar cachectic states of the constitution, and the same mode of treatment is applicable to both : for practical purposes, therefore, they may be viewed as one disease. Indeed it is often quite impossible to draw any strong lines of discrimination between some cutaneous affections, the interval between them being quite hazy and indistinct.

With respect to zona, we have no doubt in saying that Rayer is wrong when he places it among the "bullæ;" it is most decidedly a vesicular disease ; and a critical reader cannot fail to be surprised at finding the author himself admitting that "zona indeed really differs from the herpes phlyctenoides *only* in the singular form it assumes," as its very name imports ; for whatever be its seat, "zona shews itself under the form of a semicircular band of more or less extent, covering part of the trunk or of a limb." Perhaps it would be strictly more correct to designate rupia and zona as bullo-vesiculous than as simply bullous or vesiculous diseases; but as the object of nosology is only to afford the convenience of an arranged dictionary or catalogue, it would not serve any practical good to establish more divisions.

*Vesiculous Inflammations.* Under this order, Rayer enumerates four genera, viz. herpes, psora, or scabies, eczema, and miliaria : Willan and Bateman's corresponding order includes seven, which are varicella, vaccinia, herpes, rupia, miliaria, eczema, and aptha.

It will be useful to discuss briefly the propriety, the comparative merits of these two classifications. Three only of Willan's genera are admitted into Rayer's order; we have already seen that he has excluded rupia.

"With regard to varicella," says he, "I admit that, of three or four varieties which this disease offers, designated by the English pathologists as *chicken-pox, swine-pox, hives,* and *modified small-pox,* one at least, the *chicken-pox,* is really vesiculous ; but certainly, the other varieties, particularly *hives,* and *modified small-pox* are constantly pustulous diseases. By this double character, varicella forms a connecting link between vesiculous and pustulous inflammations. At liberty to place it in either of these groups, I prefer classing it among the pustulous, thus connecting it with variola, of which it may, perhaps, be a modification." 68.

These remarks have acquired more credit since Professor Thomson of Edinburgh and others have published their speculations, as to the identity of chicken-pox, with modified small-pox. It seems to us utterly impossible to decide whether we are to view these diseases as more strictly vesiculous or pustulous; the decision will depend altogether upon the period of the eruption at which we draw our description ; fortunately it is of no moment in practice. Nearly the same may be said of vaccinia or cowpox.

The genus, aptha, has, it must be confessed, no business among cutaneous diseases, unless indeed we were to admit all other affections of the mucous membranes. The only other disease in Rayer's catalogue which we have not yet alluded to is psora or scabies : and with respect to it we may with perfect propriety say, that medical men may please their fancy where they place it, and whether they call it a vesicular or a pustular disease ; for even Bateman acknowleges that "from its affinity with three orders of eruptive appearances, pustules, vesicles, and papulæ, it bids defiance to any attempt to reduce it to an artificial classification." On the whole, however, it is more correct to call it a vesicular than a pustular eruption; and the description given by Willan and Bateman will assuredly bring their readers to this conclusion; for we are told that even in the scabies papuliformis "the unbroken elevations, when carefully examined, are found to be vesicular and not papular." Such are the differences between the divisions of the order vesiculæ proposed by the English and French nosologists; the three diseases, of herpes, miliaria, and eczema, are common to both systems. From what has now been said, it may be deduced as a very fair and rational inference, that it would perhaps be wiser to employ a general term, compounded of the two terms, for the two orders of skin disease, viz. the pustular and the vesicular, and to distinguish them into the pustulo-vesicular and vesiculo-pustular, according to the predominating character of the eruption. The only member of the family which might be refractory, and to which we should be obliged to use a rather compulsory mode of introduction is miliaria; for, strictly speaking, it is vesicular, or at least very nearly so, throughout its course. It may be said likewise that small-pox is genuinely and uniformly pustular; it is so certainly in every case during its progress; but it is very common to observe numerous distinct vesicles interspersed with the papulæ when the eruption first appears.

The only remark which we shall make on the vesiculous diseases of our author respects the description given of the miliaria, (which it is to be remembered includes the sweating sickness of the 16th century, and the epidemics of the "suette miliare," which have occurred of late years in some of the French provinces.) He says that "it is an acute contagious disease, affecting at the same time the gastro-intestinal mucous membrane and the skin," and yet we are informed in the next page, "that several physicians have inoculated themselves with the matter of the vesicles with impunity." The idea of any contagious property is quite chimerical, and has arisen from the disease, at least one form of it, appearing as an epidemic; indeed so far from miliaria being generally contagious, it may be very often induced artificially, especially in plethoric and irritable subjects, by confinement in heated chambers, and the use of hot stimulating diluents; hence it used to be common about sixty years since among puerperal patients; and now it is very rarely met with. The epidemic form is not known in this country; yet it may be interesting to learn its characters from our author, who published an account of it, as it appeared in the department of the Oise, during the year 1821. He says—

"In the epidemic which prevailed in the department of the Oise, 1821, several individuals who retired to bed in perfect health, on awaking, found themselves attacked by the disease, and were inundated with sweat, which continued till death or convalescence took place. In some instances, a scarcely sensible febrile action, a burning heat, or a creeping sensation running through the limbs, with almost always a sense of constriction in the epigastrium, preceded for several hours the appearance of the sweat, or rather of a hot vapour, which at first confined to certain parts of the body, afterwards extended over the whole surface. The mouth was clammy, covered with a dirty-white coat, rarely yellowish; there was little or no inclination for food; urine natural. The bowels were constipated during the whole course of the disease, in general. In most cases the pulse was natural, but became frequent when the eruption appeared. Respiration was attended by that kind of embarrassment which takes place when the temperature is too high. The encephalon, the organs of sense, and those of generation, were not included in these derangements. This state continued, with slight variation, the second, third, and fourth days of the disease. On one of these days, and usually the third, after slight smarting, the eruption appeared, first on the sides of the neck, on the nucha towards the ears, and under the breasts in women, then on the back, inside of the arms, lower part of the abdomen, and inner parts of the legs and thighs.

Miliaria may be general and rapid, partial and slow, circumscribed and ambulant, extensive and spreading, distinct or confluent. The vesicles which characterise it are about the size of a millet-seed, pearly and diaphonous, more distinct when the skin is put upon the stretch and looked at obliquely, and are perceptible to the touch. These vesicles are often interspersed by red inflamed papulæ, which render the skin irritable; lastly, true bullæ may be developed accidentally on different parts of the body.

The duration of the vesicles is two or three days. Then they dry, and are followed by a more or less considerable desquamation. This vesiculous inflammation is attended by an abundant fœtid sweat, having a similar odour to that disengaged from rotten straw. It appears at the commencement of the disease, and is continually exhaled, under the form of a dense steam, its whole duration." 96.

He adds—

"It is seen only between the forty-third and fifty-ninth degrees of latitude. Humid and shaded situations favour its development; but it spreads, like measles and scarlatina, to the most elevated places." 97.

*Pustulous Inflammations.* It is unnecessary, after what we have stated above, to enlarge upon the general features of this order of cutaneous affections; we shall therefore content ourselves with merely pointing out the leading differences between the arrangements of Willan and Rayer. The one enumerates five pustulous diseases, viz. impetigo, porrigo, ecthyma, variola, and scabies; the other subjoins to the four first, (scabies being considered vesiculous,) varicella, vaccinia, vaccinella, cuperosa, (including the varieties of acne,) sycosis and artificial pustule. The following epitome of the author's reasons of dissent from the arrangement of his predecessors is brief and satisfactory.

"I have already remarked, that in Bateman's classification, psora has been erroneously placed among the pustulous diseases, and I have explained the motives which have led me to place variola with varicella, three varieties of which are indisputably pustulous. Vaccina and vaccinella ought to be placed in the same class, and not with vesiculous diseases. Indeed, pustules differ from vesicles, not only in containing pus or non-serous humour, but by the depth and intensity of the inflammation. This latter point appears the more important, as the serosity of all vesicles becomes turbid and purulent in the stage of desiccation, and the contents of all pustules are at first serous. Lastly, Willan and Bateman were deceived when they supposed that cuperosa and mentagra were announced by tubercles, for these inflammations are primarily *pustulous*." 99.

Vaccinella, or modified cow-pox, has hitherto been scarcely recognised by English writers; and as it is of high importance at the present time, when serious doubts are entertained by so many of the protecting efficacy of vaccination, to arrive at some satisfactory conclusions, it cannot fail to interest our readers to be made acquainted with the sentiments of foreigners. The extract is long, but very valuable.

"I designate under the name of *vaccinella*, a pustulous phlegmasia of the skin, which the insertion of vaccine virus, of *cow-pox*, or of *grease*, sometimes produces in individuals who have previously had small-pox. It may be also produced by the inoculation of variola and vaccina closely following each other; it is occasionally developed in man by the inoculation of spurious cow-pox. This disease bears the same relation to vaccina that varicella does to variola.

*Vaccinella produced by the inoculation of vaccina in individuals previously variolated.** When those who have been the subjects of small-pox are vaccinated, it usually is not productive of any result, and the punctures soon heal up; but sometimes a *vaccinal eruption* is developed, *modified*, both in its external characters and progress. This cannot be better exhibited than in those cases of varicella which the insertion of variolous virus produces in some vaccinated individuals; or than in those cases in which inoculated or variolated individuals are submitted to the influence of a new inoculation of variolous matter. Whichever of these takes place, the progress of this modified vaccina is as follows:

From the first to the third day the punctures inflame; pustules form, generally resembling those of vaccination. Their edges are flat, unequal, and not distended by the contained humour, which is always of a limpid yellow, and small in quantity. The areola, at times pretty vivid, is rarely so large as in vaccina, and is later in its appearance. During this stage, the patient usually experiences an insupportable itching in the punctures; the axillary glands become enlarged and painful, cephalalgia supervenes, and sometimes irregular accessions of fever. The inflammatory stage is very rapid; there is neither tumour nor circumscribed induration, as in vaccina; and if there is tension about the wound, it is irregular and superficial. The crusts, well formed about the seventh or eighth day, do not fall so soon as those of vaccina; they occasionally present the same appearance, with this only difference, that they are not so large, or thick, and leave no cicatrix, but only a kind of spot on the skin.

2°. *Vaccinella produced by the accidental insertion of cow-pox in a subject who had been variolated.* Jenner† reports that he saw in a farm, five persons who had previously had small-pox, contract vaccina, by coming into contact with cows suffering under the disease; but he adds, that it was incomparably more benign than in ordinary cases.

3°. *Vaccinella from the inoculation of grease, in a variolated subject.*‡ At the beginning of the year 1801, Mr. Loy saw an eruption on the hands of a farrier, who had previously had small-pox. This eruption appeared not long after the man had dressed a horse affected with *grease*. It consisted of distinct, round pustules, containing a limpid fluid, resembling the vesicles of a burn, having in their centre a small black spot, and which were surrounded by an areola. In the whole duration of the eruption, no fever was manifested.

4°. *Vaccinella from the inoculation of variola, a short time subsequent to vaccination.*|| When the variolous and vaccine virus are inserted at the same time, they reciprocally modify the action of each other. The vaccine pustule thus produced is smaller than usual, its progress more tardy; the areola scarcely perceptible, or formed prematurely. On the other hand, the variola is altered, and it appears under the form of hard, glistening pustules.

5°. *Vaccinella produced by spurious cow-pox.*§ 'We frequently see,' says Jenner, 'ulcers and pustules spontaneously developed on the teats of cows; par-

---

* *Rapport de la Commission Medico-chirurgicale Instituée à Milan.* Paris, an. x.

† Jenner, *An Enquiry into the Causes and Effects of the Variolæ Vaccinæ.* 4to. London, 1793.

‡ Loy, *Account of Experiments on the Origin of Cow-pox,* 1802.

|| Willan, *On Vaccine Inoculation,* 4to. Lond. 1806.

§ Jenner,—Tellegen, *Dissertatio de Variolis Vaccinis.* Groningæ, 1801.

ticularly in the spring, when they change their food. These pustules may be transmitted to those persons employed in milking; but they are always of a much more benign character than those of true *cow-pox,* and do not protect from variola.'

Of all these eruptions, one only has been well studied, viz. that produced by vaccination, in previously variolated individuals. The characters which distinguish vaccina from vaccinella have been already described (§260.) These also differ from the accidental pustules which have been denominated *false vaccina,* or *false variola* (according as they have been produced by pus taken from vaccinated or variolated subjects), by their progress, and umbilicated form.

Other varieties of vaccinella have been rather indicated than described, and their history requires to be duly studied." 129.

No pustular diseases are so difficult to describe, and accurately to discriminate, as the varieties of impetigo and of porrigo, or, as Rayer calls it, tinea. The portion of Willan's work which treats of them is decidedly one of the most faulty, and some of the errors which he has committed in his arrangement cause, unfortunately, a direct and practical mischief. The distinction which he has made between them is founded upon the supposed contagion of the one set, and the non-contagion of the other; had he been able, indeed, satisfactorily to point out the diagnostic characters of each, the distinction would be one of the most useful that could be adopted. Impetigo he defines to be an "eruption of non-contagious psydracia, occurring chiefly on the extremities, and without fever." He enumerates five species—imp. figurata, sparsa, erysipelatodes, scabida, and rodens. Rayer remarks, and we quite agree with him, that "Willan has made too many varieties of impetigo: his *I. scabida* should be joined with *I. figurata; I. erysipelatodes* is nothing but the eczema *impetiginodes* of the same author; lastly, *I. rodens* is really only a variety of cancerous ulcer, the edges being covered by accidental psydraceous pustules." But a much more grave objection seems to be, the omission of the disease long known under the names "crusta lactea," or "milk scall," from the order impetigo, and its insertion in the order porrigo. Bateman has, indeed, pointed out this error, and proposes to call it "impetigo larvalis;" but, with most injudicious devotion to his master's precepts, he has retained it among the species of porrigo; the characters of the eruption, as well as the non-contagion of the disease, ought to have determined him to make the necessary change. Rayer, too, has foolishly followed his predecessors, and has introduced not a little confusion himself into his order "tinea," which corresponds to Willan's "porrigo." Indeed, there is no order of cutaneous affections which stands so much in need of a thorough revisal as this; Willan's arrangement is bad, and Rayer's is not much better—for, not to mention the "porrigo larvalis" of the one, and the "tinea mucosa" of the other (the same disease, be it remembered), which, as we have already said, is decidedly an impetigo, the species P. furfurans, and decalvans, and perhaps, also, the favosa, are of most questionable admissibility; and, to make things worse, the French nosologist applies the specific names used by Willan to different species, as we shall presently shew.

Willan enumerates six species, viz. P. larvalis, furfurans, lupinosa, scutulata, decalvans, and favosa. It is very doubtful whether the P. furfurans is really contagious; and the circumstances under which it usually appears, viz. at the termination of the "P. scutulata," or genuine ringworm, when the pustulous disease is subdued, but before the skin has returned to its healthy condition, and, moreover, the resemblance which it has, after some continuance, to the P. decalvans, should forbid, in our opinion, its insertion in the genus at all. With respect to the latter species, or "decalvans," we cannot do better than extract the following passage from Rayer.

"One of the most remarkable varieties of alopecia (or baldness) is that which Bateman designated under the improper name of porrigo *decalvans*. The scalp of persons affected with it presents one or more circular patches entirely bald in the midst of a luxuriant head of hair. The skin is shining without being red, and is often remarkably white. The area of these circular spots, progressively increases. If several exist close together they unite, and if left to itself the disease may affect the greater part of the scalp. Bateman supposed that small pustules were developed at the root of the hair, but acknowledges never to have seen them. I am, myself, ignorant of the nature of the affection of the pilous follicles in this variety of baldness; I can affirm, however, that there does not exist, on the surface of the scalp, either vesicles, pustules, or any other form of phlegmasia; the skin is always colourless. The new hair growing on these surfaces is, in general, of a finer texture and lighter colour than the original. It is generally grey in adults. I have observed this affection both in children and adults, but cannot point out its causes of development." 365.

Moreover, there are no established proofs of its contagiousness. We have thus dismissed three of Willan's six species, and are inclined to extend the 'congé' to a fourth, viz. the P. favosa. It is certainly much more closely allied to the "P. larvalis," or "crusta lactea," than to any of the other species enumerated by Willan; this, of itself, will make us rather sceptical of its truly porriginous character; and what may well add to our doubts is, the circumstance of Bateman, in the description which he gives of it, never once alluding to its contagious property; and, again, the analogy which exists between it and some of the pustular cutaneous affections, which we witness in the secondary stages of syphilis, especially after the injudicious exhibition of mercury, may be adduced as a most staggering argument. The other two remaining species of porrigo we shall leave 'intact,' and now proceed to make a few remarks on the division of the genus "tinea," by Rayer.

"I am induced (says he) to admit four species of tinea: *T. favosa, T. annularis, T. granulata,* and *T. mucosa.* I must premise, however, that I regard these four pustulous inflammations as perfectly distinct from each other, and not as species or varieties of the same disease. Their individual existence rests on characters as marked as those of any other pustulous inflammation of the skin. Of the tinea, some are contagious, others not; this alone destroys all supposition of the identity of the nature of these diseases. Lastly, they can still less be considered varieties of the same affection, from the fact of their being found complicated with one another. I use the generic term of *tinea,* only because it has been so long in use, and not as indicating any identity between diseases which does not really exist." 151.

It is to be regretted that he does retain the generic term of tinea, after having given such satisfactory reasons for expunging it. The T. mucosa is, as already mentioned, the same disease as Willan's porrigo larvalis. The T. favosa includes Willan's P. favosa and P. lupinosa; the description of it is perplexingly confused, and, if we are not much mistaken, this confusion arises from two very distinct affections being blended together; the definition given is "a chronic contagious inflammation of the skin, characterized by *very small pustules*, the summits of which soon become converted into yellow, very adherent crusts, depressed into a cup-like shape;" and yet there is repeated mention afterwards of "favous pustules," which, in the usual acceptation of the term, are *not very small pustules*. If, by "T. favosa," we are to understand the disease which, by Cullen and others of the olden times, was called tinea capitis, or scald head, then assuredly it must be very materially altered in its characters. According to their descriptions, the pustular eruption was confined to the hairy scalp, and did not extend "to the shoulders, arms, trunk, and lower extremities," as stated by Rayer, who tells us that he has seen it on these parts, while the scalp remained unaffected. But we put the simple question—is the eruption on the arms and legs really and truly contagious? We think not; and, if our opinion be correct, then it must be confessed that two diseases have been confounded together. The subject is one of great practical importance, and ought to be diligently studied by those who have extensive opportunities of observation.

As to the other two species of Rayer, the "T. annularis" and "T. granulata," they correspond to Willan's single species, P. scutulata. Our limits prevent us from alluding more particularly to the characters of each; and we regret this the less, as we are ourselves quite bewildered in the meshes of incongruous narratives, and do not feel ourselves warranted to attempt the unravelling of them. Our readers, however, may receive considerable instruction from our review of Mr. Macilwain's treatise on Porrigo, in our Number for last April. Passing over the order of "furunculous inflammations," which have not usually been recognized as strictly cutaneous diseases, we come to that of the—

*Papulous Inflammations*—the "papulæ" of Willan. Both systems admit three species, viz. strophulus, lichen, and prurigo; but, as Rayer observes, "they might be reduced to two—strophulus appearing to be but a modification of lichen, in new-born infants and those at the breast." The itching, however, is never so considerable in the former as in the latter, although the skin of the child is naturally much more irritable than that of the adult. Our author follows very closely the descriptions given by Willan and Bateman; it is, therefore, unnecessary to dwell upon this order of cutaneous affections.

Before leaving it, we may repeat that it would have been more consistent with the principles of philosophic arrangement to treat of papulous, before pustulous and vesicular eruptions. Rayer ought to have followed his English guides in this particular. The next order is that of—

*Tuberculous Inflammations.* This order of Willan has been subjected to a most unmerciful pruning, two only of the original genera being retained, viz. lupus and elephantiasis (and only one form of it); and to these two, has been added, "cancer or scirrhus of the skin." The definition indeed of a tubercle is vague, and almost unintelligible; and perhaps we could not find a more satisfactory proof of the extreme difficulty of the classification of living phenomena than by referring to this department of our present inquiries. The genera of "lupus" and "cancer," though indisputably affections of the skin, may for any practical uses, be excluded from a treatise on cutaneous diseases; they are very rarely submitted to the inspection of a physician, and the most modern surgical works have treated of them at great length: and with respect to the other genus, "elephantiasis of the Greeks," it was our intention to have discussed at some length the characters of this, as well as of the Arabian elephantiasis, or as it is called in the West Indies, the Barbadoes leg, and to have pointed out the differences between these two diseases, and also between them and the Jewish leprosy or leuce, a disease which has been so often confounded with them; for, indeed, on no one question of medical inquiry has there been so much discreditable ignorance displayed, and such astonishing absurdities committed, as in the histories of these affections: we had wished to have swept away some of the rubbish in which the subject has been involved; but for this we cannot find room at present; and, although interesting, it must be confessed that it is one on the whole rather of literary curiosity than of directly practical importance to the medical man. We shall only remark that in our opinion Rayer has not done well to separate the elephantiasis of the Arabs from all other diseases of the skin, and to have made a distinct section for it alone, on the ground of it being "a disease primarily foreign to, but which, at times, produces alterations of the skin." Is it primarily more foreign to the skin than boils and carbuncles are? We think not. The order of "squamous inflammations" follows. Rayer omits icthyosis, "which has," he says—

"But small analogy with squamous diseases. Willan and Bateman have thought proper to place them in the same class. Icthyosis, almost always congenital, or developed in the first few months after birth, is attended neither by sanguineous injection of the skin, morbid heat, itching, nor any other sign of inflammation. In lepra, psoriasis, and pityriasis, the production of the scales is always preceded by redness of the skin, which may be made apparent by removing them from its surface. In confluent and inveterate lichen, developed on the trunk or limbs, the skin may become brownish, and covered by innumerable scales, somewhat similar to those of slight icthyosis; but this state is accompanied by insupportable itching, and preceded by papules. The simultaneous or ulterior development of similar elevations, upon some adjacent part of the skin already farinous, will dissipate all doubts as to the nature of these obscure cases." 337.

Although correct in the main, these reasons do not appear to us quite sufficient to have warranted the expulsion of icthyosis from the order of the squamæ: the utter absence of inflammatory action, or at least of something approaching to it during the early stages, is problematical; for the history of the disease has not been hitherto examined with sufficient accuracy.

The ninth and tenth orders need not detain us a moment, and we therefore proceed at once to the last or eleventh, in which are included several most incongruous affections; our business shall be, however, only with one set of them, which may be well called multiform (provided we are to adopt as the generic term, syphiloid disease, or syphilitic eruption) as there is scarcely a form of cutaneous disease which it does not assume. It appears sometimes as an exanthema, (roseola syphilitica), characterised by broad patches of a coppery red colour, scattered over the trunk and limbs; sometimes as a papular, (lichen syphiliticus); or as a pustular or tuberculo-pustular; and still more frequently as a squamous eruption (lepra syphilitica). Now all these eruptions have one outward feature in common, a feature too not observed in other diseases, and which therefore serves to enable us very generally to pronounce on the nature of the case; this feature is the coppery or violaceous red color which pervades and surrounds the cutaneous blotch, of whatever character the blotch may be; in addition to this feature, there is another, which though not so universal is so very commonly present, and so pathognomonic, that when it does occur, our diagnosis is rendered almost infallible : the papules, pustules, tubercles, and scaly spots, (for the remark is not applicable to the venereal exanthema) have all a tendency, when left to themselves, to terminate in ulcers, " the edges of which are irregular and sharp, while the bottom is unequal and of a greyish white colour." These different forms of eruption are not unfrequently seen on the same individual at the same time; or one form may be developed, as another fades away. The most frequent and the least equivocal form is unquestionably the leprous, and as it is of great importance that the disease be speedily recognized, we direct the attention of our readers to the following very accurate description of its appearances.

"The patches are ordinarily dry, flat, circular; from four to six lines in diameter, and of a *coppery red colour*. Each spot is announced by a small hard elevation, red or violaceous, which extends circularly till it reaches its fullest dimensions. These plates or patches, very distinct and separate from each other, are always of a copper tint when arrived at their highest degree of development. They scarcely exceed the level of the skin; their surfaces are, at first, as soft and smooth as the healthy skin; they afterwards become scaly or furfuraceous, but the copper colour always decides their syphilitic nature. Sometimes the centre is paler than the circumference; at times they are formed in circles or rings, in the centres of which the skin remains perfectly healthy; but still, the peculiar copper tint does not allow of their being confounded with lepra or psoriasis.

Left to themselves, these patches run on to ulceration, after the development of a small psydraceous pustule. The light scales which cover their surfaces become detached, and are replaced by others of greater thickness; a small crust is then formed, beneath which ulceration takes place, but rarely to any extent, and generally superficial." 268.

The pustular form of syphilitic eruption is not so uniform in its appearances as the leprous. The pustules may be either large or phlyzacious, small or psydracious, or tuberculoid : the first of these varieties constitutes the "ecthyma syphiliticum," of some authors; and perhaps most of the cases of Willan's "ecthyma cachecticum," are in truth attributable to the action of the venereal, or of the mercurio-venereal poison, on an unhealthy constitution. After continuing for some days, these (the phlyzacious) pustules give issue to a purulent fluid, which concretes under the form of blackish-brown crusts, usually conical and surrounded by a copper-coloured areola; when these crusts fall off, there are observed either brownish spots, or small ulcers, or cicatrices of such ulcers, all presenting more or less the same hue. Not unfrequently numerous papulæ are intermixed with the pustules, and then we have a compound or papulo-pustular eruption. Our author's description of the tuberculoid form is good.

"*Syphiloid tubercles* are ordinarily observed on the alæ of the nose, commissures of the lips, on the forehead, genitals, &c. They are also seen on the limbs. These tubercles vary in size from that of a cassia-seed to that of an olive; they are round or ovoid, scattered or disposed in groups, and at times symmetrically arranged one after the other. Their livid or copper colour, contrasts singularly with that of the surrounding healthy skin. Their surfaces are at first smooth and shining; but when they have been long neglected, they inflame and *ulcerate*. Among the ulcerations, some are covered by thick adherent crusts, frequently conical. When these become detached, the ulcer sometimes heals at its centre while it is spreading at its circumference; and if the patient is much debilitated, the ulceration acquires considerable dimensions. Tubercles may also terminate in *serpiginious* ulcers, healing on the one side, and spreading on the other. These deep and sinuous ulcers, spirally disposed, resemble ciphers, letters, segments of circles, entire rings, &c. the livid edges of which are sometimes surmounted by tubercles. After their cure, they leave indelible cicatrices on the skin, of a very irregular form." 271.

The syphilitic lichen is characterised by the eruption of papulæ, which soon acquire a coppery color, and which are usually surrounded with areolæ of the same hue. If neglected, these papulæ are apt to ulcerate, and "like other forms of syphilis, this eruption is successive in its development; and on the same subject, and sometimes on the same region, may be observed papules intact and ulcerated, and small circular cicatrices, having irregular edges, while their centres are depressed." Of all the forms of syphilitic eruption, the pustular and tubercular are the most serious, not only as indicating a more vitiated state of the constitution, but also as being much more difficult of cure, and requiring an especial nicety of discrimination in the treatment. As a general remark, they are made worse by severe courses of mercury, and almost inevitably, if there is induced a profuse salivation. The exhibition of small alterative doses, in conjunction with the use of sarsaparilla, a nutritious diet, and with change of air, is the most successful, as well as the safest practice to pursue. But this branch of the subject, although the most immediately and practically interesting, we have not space to discuss; our article having already exceeded the limits which we wished to prescribe to ourselves at starting. A third part at least of the volume remains unreviewed, although containing much curious as well as useful information; the nature of its contents may be gathered by an inspection of the synoptical table which we have given. Some may have thought that we have been too much of mere critical nosologists throughout this article, and may object that, scarcely one remark has been offered with respect to the treatment of any of the maladies which have passed under review. We have a reason for having acted so; and it is this. Rayer's treatise is as a whole, a work of classic excellence; it is valuable especially for the accurate descriptions of symptoms and of appearances, and for the minute attention to the diagnosis of cutaneous diseases; but the therapeutic portion is decidedly much inferior to the descriptive; and high though its general merits be, we should be wrong in withholding our opinion, that in its present form it is not all adapted to supersede Bateman's admirable Compendium. Perhaps with the exception of having pointed out, with greater earnestness, the efficacy of bloodletting in many forms of cutaneous disease, the author has made scarcely one valuable suggestion touching the employment of the most approved remedial means. We must not therefore consult this work alone, in guiding our practice in troublesome and perplexing cases, or we shall be grievously disappointed: Willan and Bateman are much better authorities. The translator might have done much, had he exerted himself to have remedied the defect to which we allude; but unfortunately he has confined himself to the mere school-boy occupation of substituting English for the French words of the original; there is not a single note of the least value appended to any part of the volume; and even this is perhaps the very smallest blemish of the book; it has been most carelessly (we suppose from the excessive haste on the part of Mr. Dickinson to see his name in print) got up, and it is full of errors, which he may perhaps say, are merely typographical, but which we are not quite willing to admit as such; for not to mention the blunders in transcribing a few German words which occur here and there, what shall our well informed readers say of such classic terms as "febra urticata," "tinea annulare," and a score of such; or of a line like the following, "de febre a caneris flaviatilibus, et fragaria vescæ fructu." ? Almost every page presents such words as "veriola," "porrigo scutelata," "venerala," "visiculous," "flour of sulphur," and so forth. The formulas for the preparation of certain ointments and lotions are quite unique; for example—

"℞. Protochl. mercury, ʒii.

Sugar, thus, āā ℥ss. M. for fumigation."

An ointment for the itch is to be prepared thus:—

"℞. Adip. ℥j.

Sulphur. ℥ij.

Potassæ, ℥j. M. ft. ung.

Two frictions, of two ounces each, twice a day."

Pray is the potassa the caustic alkali? We pity the poor patient, if it is; we should think that the fate of Marsyas, when he was flayed by the wrathful god, was not much worse. Then for another specimen. ("Redwash Hospital, St. Louis.")

"℞. Deutochl. mercury, ʒj.

Distilled water, ℈j.

Anchusa, q. s. M. ft. lotio."

A drachm of corrosive sublimate to a scruple of water! A moderately strong wash we suppose. Dr. Paris, in the next edition of his Pharmacologia, will do well to attend to these curiosities of the prescribing art. In the event of a second impression being at any future time called for, Mr. D. will do well to revise studiously and patiently the work which he has undertaken to introduce into English medical literature. Let him not be satisfied with giving a mere bald translation of Rayer's text; he should aspire to a more dignified avocation; and by comparing the sentiments of his author with those of domestic and of other continental writers, by abridging or altogether excluding some of the unnecessary details, and by supplying the deficiencies, (more particularly in reference to the treatment of skin diseases) he will not only do himself an essential good, but confer a practical benefit on the profession at large. As a guide and pattern for him to follow in his labours, we cordially recommend him to take Dr. Forbes, whose translation of Laennec we cannot praise too highly. A copious index is indispensably requisite in such a work as Rayer's.

